# SEM Image Analysis in Permeable Recycled Concretes with Silica Fume. A Quantitative Comparison of Porosity and the ITZ

**DOI:** 10.3390/ma12132201

**Published:** 2019-07-08

**Authors:** Manuel J. Chinchillas-Chinchillas, Carlos A. Rosas-Casarez, Susana P. Arredondo-Rea, José M. Gómez-Soberón, Ramón Corral-Higuera

**Affiliations:** 1 Faculty of Engineering Mochis, Autonomous University of Sinaloa, Fuente de Poseidón y Ángel Flores s/n, Col. Jiquilpan, Module B2, Los Mochis, Sinaloa 81210, Mexico; 2 Barcelona School of Building Construction, Polytechnic University of Catalonia, Av. Doctor Marañón 44-50, 08028 Barcelona, Spain

**Keywords:** porosity, recycled aggregates, permeable concrete, interfacial transition zone, image analysis, porosimetry mercury intrusion

## Abstract

Recycled aggregates (RA) from construction and demolition can be used in permeable concretes (PC), improving the environment. PCs have a significant porous network, their cement paste and the interaction between the paste and the RA establishing their strength. Therefore, it is important to evaluate the porosity in the interfacial transition zones. The porosity of the cement paste, the aggregate and the interfacial transitional zones (ITZ) of a PC with recycled coarse aggregates (RCA) and silica fume (SF) is measured by means of image analysis–scanning electron microscope (IA)-(SEM) and by mapping the chemical elements with an SEM-EDS (energy dispersive spectrometer) detector microanalysis linked to the SEM and, as a contrast, the mercury intrusion porosimetry technique (MIP). In the IA process, a “mask” was created for the aggregate and another for the paste, which determined the porosity percentage (for the anhydrous material and the products of hydration). The results showed that using SF caused a reduction (32%) in the cement paste porosity in comparison with the PC with RA. The use of RA in the PC led to a significant increase (190%) in the porosity at different thicknesses of ITZ compared with the reference PC. Finally, the MIP study shows that the use of SF caused a decrease in the micropores, mesopores and macropores.

## 1. Introduction

PC is a special concrete with a high gap content and high permeable capacity when compared with conventional concrete [1,2]. This PC offers several environmental benefits, among which are rainwater runoff control, replenishment of underground water supplies, an improvement in water quality and a reduction in water and subsoil contamination [3]. Therefore its use is habitual in parks, reduced traffic areas, tennis courts, etc. [4]. This PC contains interconnected macropores of sizes that oscillate from 2 to 8 mm and with gap content of between 18% and 35%; therefore, its mechanical strength is closely linked to the strength of the cementing matrix itself and the interaction of the matrix with the aggregates that form it. Consequently, it is necessary to determine and study the porosity of these areas of interest [5].

The use of natural aggregates is currently widespread in the manufacture of PC [6], but few studies have used RA [7]. As important quantities of construction and demolition waste are contaminating many ecosystems worldwide, it is imperative to recycle them and produce new building materials, recycled concrete [8]. The production of RA from construction and demolition waste has been studied widely in the last two decades and the studies agree that these aggregates are more heterogeneous, less dense and more porous than the natural aggregates [9,10]. Those studies which have analyzed the microstructure of the RA show that there are two ITZ a) between the old natural aggregates and the old matrix of the cement and b) between the RA and the new cement matrix [11]. The ITZ is considered by many researchers to be the weak link in the concrete [12,13], in which the normal concrete fails, significantly compromising both mechanical properties and permeability; this is also characterized by being an area of high porosity [14]. This differentiated zone can form due to the “wall” effect caused by the looser packing of the cement granules against the surface of the aggregate, or because of the accumulation of free water around the surface of the aggregate [15]. Some models of the ITZ have established that it is a space with ‘uniform’ porosity, located 5–30 μm from the aggregate surface; in other models, it has been found up to 50 μm from the aggregate [16]. Porosity in the ITZ is one of the basic factors influencing the strength and durability of concrete; the more porosity in this zone, the less mechanical strength and the greater the vulnerability to environmental degradation [17]. Additionally, the porosity in the cementing matrix can be determined as the sum of the volume of the capillary pores, the ice pores, the macropores formed by the presence of air and macropores caused by compacting [18]. This porosity can also be considered as the spaces not filled by the solid components of the hydrated cement paste, which depend to a great extent on the ratio of water/cement (w/c) and the degree of hydration reached by the cement [18].

Nowadays several alternatives that allow a reduction in the porosity percentage in concrete have been established, such as the inclusion of additives, the setting of low w/c ratios, the inclusion of fibers or the application of pozzolans [19,20]. Regarding the last alternative, SF is the most used pozzolan for improving the properties of concrete and reducing the porosity [21] when the ITZ is densified; this can react with the cement hydration products (calcium hydroxide—CH–) to form calcium silicate hydrate (CSH), which thereby improves the mechanical strength. Similarly, this SF also acts as a filler which reduces porosity, as the granule size of this material is between 0.1 and 0.2 μm [22].

Having seen the importance of porosity in the binding matrix of concrete and in its ITZ, several studies have used various methods to try to establish and calculate the percentage of porosity. Among them are computerized tomography X-ray scans [23], mercury intrusion porosimetry technique (MIP) [24,25], mathematical models of finite elements [26] and X-ray microtomography [9]. However, the IA technique using software is noteworthy, as the treatment of images obtained from IA-SEM is a non-destructive, precise and low-cost method [27]. This method breaks the image down to a scale of gray tones to obtain a binary image of the area of interest; it is also applicable when the aggregate or cement paste is made up of various phases, making it possible to identify the hydration products such as CSH, CH or ettringite [28]. The IA makes it possible to make a mask—a selection filter—of the aggregate or the cement paste, allowing these elements to be distinguished, then establishing the porosity with exactitude [29]. The importance of this technique is reflected in those studies where the porosity of the ITZ is calculated at different thicknesses of the aggregate [28], quantifying the percentage of pores and micro-cracks in the concrete [30]. This leads to the identification and quantifying of the hydration products and the anhydrous materials of the concrete [31,32]. However, the majority of these studies have focused on conventional concretes and there is no evidence of studies of image analysis in PCs. Therefore, this study aimed to evaluate and correlate the microporosity of PC made with RA and SF using MIP and the IA of SEM; this was done through studying the porosity of the aggregates, the porosity of the cement paste, the percentage of hydration products, the percentage of anhydrous materials and the percentage of porosity of the ITZ at different zones of the aggregate.

## 2. Materials and Methods 

### 2.1. Materials

CEMEX^®^ brand composite Portland cement (30R) [33] was used to prepare the PCs. Local companies supplied the distilled water, the natural coarse aggregate (NCA) and the SF. The RCA was obtained by means of grinding waste paving concrete, with a strength of 23 MPa, in a jaw crusher in the sustainable materials laboratory of the Universidad Autónoma de Sinaloa (UAS), Mexico.

The properties of the NCA and RCA are shown in Table 1; these were determined from the ASTM C127 and ASTM C29 [34,35] standards. It should be mentioned that the maximum size of the aggregates was 12.7 mm, obtained after sieving. Of the studied properties, the high absorption of the RCA—more than 3.5 times that of the NCA—is noteworthy. In order to determine the chemical compounds present in the cement and the SF, an X-ray fluorescence analysis was carried out with a set of ARL 8680 (ThermoFisher Scientific, Waltham, MA, USA.), using the wave dispersion method according to the ASTM C114 [36] standard (see Table 2). Both the ASTM C150 cement [33] and the SF (ASTM C1240) [37] showed typical percentages of compounds. Finally, the physical properties of the Portland cement and the SF are to be found in Table 3, establishing that both are within the specifications [33,37].

### 2.2. Design of the Permeable Concrete Mixture

**Figure 1:**
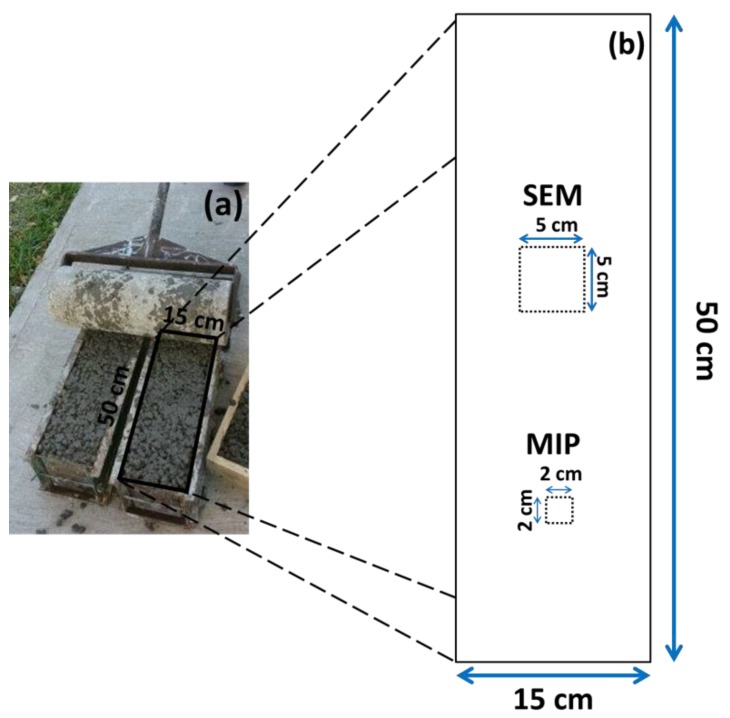
General scheme of the test samples. (**a**) real photo of the specimen beam and (**b**) areas of study for analysis of scanning electron microscope (SEM) and mercury intrusion porosimetry (MIP).

Table 4 shows the components and quantities of the different materials that make up the four dosages of the PC mixtures to be used in this study. The criteria of the RCA content percentages have been established with reference to previous similar research [38,39], as well as in the case of SF (10%) [40,41]. The values shown are those needed to make a beam of 15 × 15 × 50 cm, from which the study samples will later be taken (see Figure 1).

The nomenclature used is classified as follows: the first three letters (NCA or RCA) refer to the origin of the coarse aggregate; the following value shows the percentage of recycled aggregate used (100% or 50%); finally the last letter indicates the cement used (C = 100% Portland and SF = 90% Portland + 10% SF). A value of 0.35 was used for the w/c ratio, as is common in this type of study [42,43]. As this was a PC, no fine aggregates (FA) were added to the mixture.

The mixing process of the PC followed the methods laid down by previously reported studies [44], as well as the recommendations for using RCA, such as the need for pre-saturation [4]. When the mixture was ready, the concrete was poured into metal 15 × 15 × 50 cm beam molds, in accordance with the ASTMC31 norms [45]. The beams were kept in laboratory conditions for 24 h before being removed from the molds and immersed in a curing tank (ASTM C192) [46], until being tested or processed.

### 2.3. Test Methods

#### 2.3.1. Mechanical and Physical Properties of the PC

After 28 days of curing, the PC beams were tested for their flexural strength, in accordance with ASTM C 348 [47], and then the compressive strength was evaluated (ASTM C-349 [48]). In addition, the water permeability coefficient was assessed (ASTM D-2434-68 [49]) with a variable charge permeameter.

#### 2.3.2. Mercury Intrusion Porosimetry (MIP)

The porosimeter used for the MIP tests was a Micromeritics Autopore IV 9500 (Micromeritics Corporate Headquarters, Norcross, Atlanta, GA, USA.), with a precision of 33.000 psi (228 MPa) and pore diameter detection capacity ranging from 0.006 to 175 μm. As there are no specific norms for concrete, the norms of ASTM D4404 (for soil and rock) [50] were followed in the test, along with the evidence from previously completed studies [51]. The test age of the specimens was 90 days; they were extracted from the PC beams (approximate volume 2 cm^3^) using a Buehler Isomet 4000 precision saw9500 (Buehler, Lake Bluff, IL, USA.), with a blade cutting thickness of 20 μm. This prevents the possible formation of microfissures during the extraction process. The test tubes were dried in the permeameter vacuum chamber for 60 min at 24 °C before the inclusion of the mercury (Hg). The Hg inclusion process was started at low pressure so that the test team could identify the biggest pores; then the pressure was increased so that the Hg penetrated all the simple pore cavities. Each test took approximately 7 h 

#### 2.3.3. Scanning Electron Microscope (IA-SEM) and Microanalysis of Chemical Elements with an Energy Dispersive Spectrometer (SEM-EDS)

**Figure 2:**
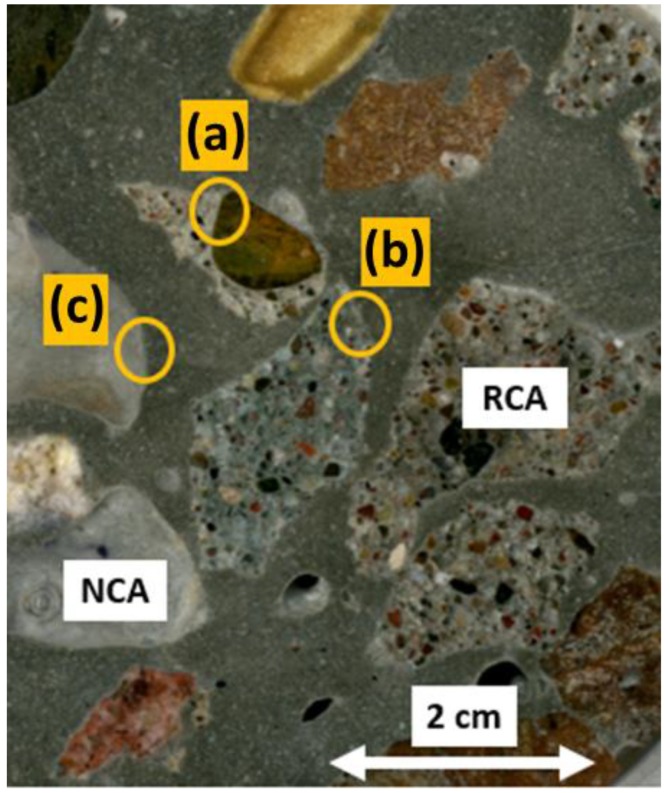
RCA 50 SF embedded in epoxy resin with a polished surface. (**a**–**c**) “areas of interest" for study in IA-SEM.

Small representative samples of the concrete beams were taken (with an exposed face of approximately 5 cm long by 5 cm wide) in order to obtain the IA-SEM images of each of the PC variables in the study. These samples were then placed within circular molds and covered with transparent epoxy resin (EpoFix Resin). They were then put in an oven for 40 min at 40 °C (catalyzer), by which time the resin had hardened and sealed the sample inside. The samples in the resin were then subjected to progressive smoothing and polishing on their exposed study face until they shone. The “areas of interest” were then chosen visually, to be studied later in the IA-SEM. As the RCA used in the PC introduced a different component to the study matrix [52,53], they were located as (a) ITZ between the new aggregate–old aggregate, (b) ITZ between new paste–old paste and (c) ITZ between new aggregate–new paste (see Figure 2); they were present in the RCA 50 SF sample. Once the “areas of interest” had been located, the samples were subjected to drying in an oven at a low temperature until they had attained a consistent weight; they were then metallized with graphite powder (obtaining a conductive surface) [54] and placed in a dryer until just before being studied in the IA-SEM.

**Figure 3:**
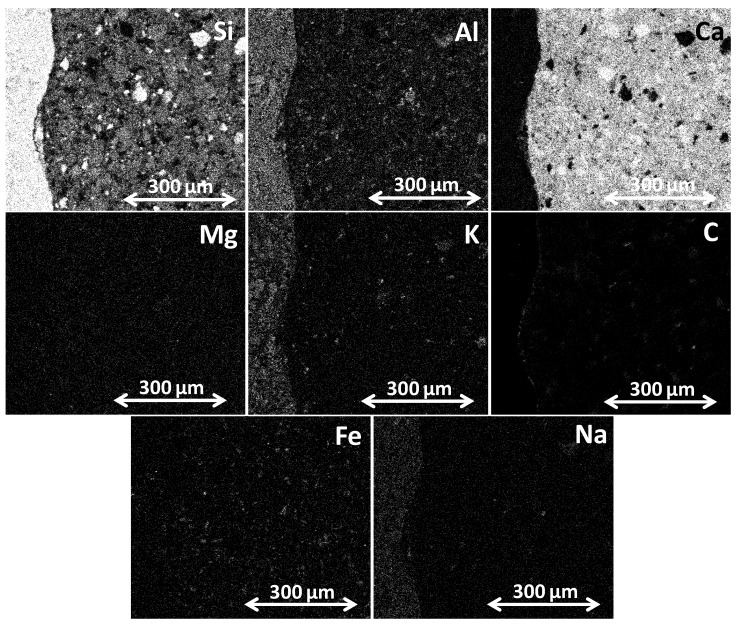
Elemental mapping of the RCA 50 SF sample between the natural coarse aggregate (NCA) and the new paste.

The IA-SEM used a JEOL JSM-6510 microscope (Jeol Ltd., Tokyo, Japan) for each image obtained from the study. The images were taken with (1) high-resolution detector of secondary electrons SEI (secondary electron image) for high-resolution images; (2) backscattered electron detector BEI (backscattered electron image) with lower image resolution but greater contrast for examining the surface topography. The zooms used for taking the images were: 70× for the general composition of the matrix, 200× for the areas of interest and 500× for mapping. The mapping involved determining the chemical composition of the samples by detecting basic elements through microanalysis, which was done with an energy dispersive spectrometer (EDS) (Inca 200, Oxford Instruments, Abingdon-on-Thames, UK) connected to an SEM unit. The EDS study required 14 complete scans for each image in BEI mode to determine the chemical elements present in the samples chosen: Si, Al, Ca, Mg, K, C, Fe and Na (typical in the chemistry of concrete [55,56]). Figure 3 shows the locations of the chemical elements present in one of the micrographs. 

#### 2.3.4. Image Analysis Using NI Vision Assistant

The image analysis process was carried out using National Instrument’s NI Vision Assistant 2018 software (LabWindows/CVI Version 7.1, Austin Texas, TX, USA.), as this has proven itself to be a useful image processing tool in industry and among the scientific community [57]. This software’s full set of processing functions and digital image capture improves project efficiency by reducing the programming effort and producing better results in less time [58]. Among this software’s functions are the study of particles, pattern recognition, detection of edges and barely visible colors, calculus of segmentation and frequency histograms [59]. Image analysis involves examining an image in detail, separating the components in order to know their characteristics and qualities and obtain results. As this software allows separate images of the PC components to be captured, it was possible to analyze the porosity and calculate the amount of anhydrous material and hydration products contained in the PC. Using operations integrated into the software (addition, subtraction, erosion, aggregation, etc.) it was possible to create the SCRIP (batch processing file or command file), which was used in treating the images, creating the masks for the aggregate and the paste.

##### Making the Masks

The procedure of carrying out an image analysis is the result of applying complex numerical algorithms that process the incidences in the pixels of an image. This was performed by the software, which controlled each numerical operation in an exact, balanced and precise manner for the specific area of interest of each image.

**Figure 4:**
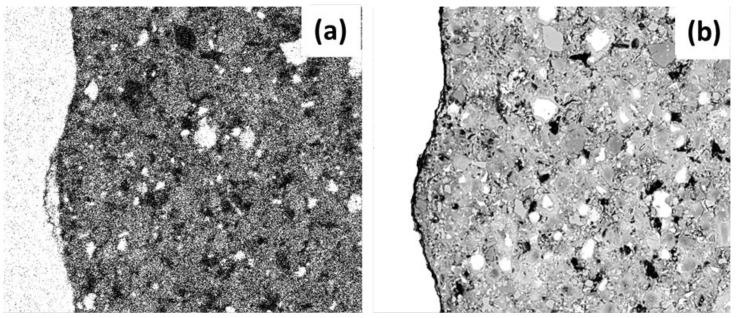
Preparation of the aggregate mask for the RCA 50 C sample (**a**) image after the operating instructions and (**b**) after the morphological filters.

The applied image analysis procedure began with the calibration of the image’s dimensions and the relationship between this and its pixels. Then, as the first step in the analysis, the aggregate mask was made. As this was composed mainly of Si, K, Na and Al (Figure 3), the “operating instructions” of NI Vision Assistant (addition, subtraction, multiplication and division, etc.) (Figure 4a) were used to form an outline image. Subsequently, morphological filtering was applied. This is a numerical process based on the definition of the connectivity of pixels. Each pixel may be connected horizontally, vertically or diagonally with its neighbors (these morphological filters may be based on a matrix of pixels with a pre-established structure, e.g., hexagonal, square, etc.). Therefore, in a morphological filter, it is possible to define several versions in order to surround the pixel to be studied [29]. This means that the process has sufficient ‘sensitivity’ to allow the applied procedure to adjust better to reality, thereby preventing any significant alteration to the original image.

In accordance with the result of the operating instructions, the morphological filters applied to the images were as follows: (a) filtering (noise eradication), (b) opening of objects (eroding and dilating the image—always with the same structural element and permitting the image to keep its original size), (c) elimination of small objects (removing those found inside larger ones) and (d) filling and closing objects (joining and harmonizing elements). The image of the aggregate (aggregate mask, Figure 4b) was established from the results of this group of filters.

**Figure 5:**
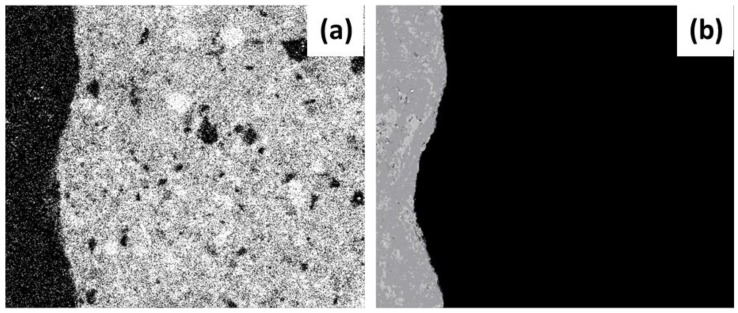
Elaboration of the mask of the paste for the sample RCA 50 C (**a**) image after the operating instructions and (**b**) after the morphological filters.

Using the same analysis procedure (operating instructions and morphological filtering), but on this occasion with the images obtained from SEM-EDS which define the basic compounds of Ca, C, Fe and Mg (incidences present in the paste), it was possible to obtain the masks that outline the paste, as shown in Figure 5. Finally, for the samples with RCA three masks were made from the aggregate and the paste in order to evaluate the porosity in different areas of the ITZ (a) new aggregate–new paste, (b) old aggregate–new paste and c) old aggregate–old paste.

**Figure 6:**
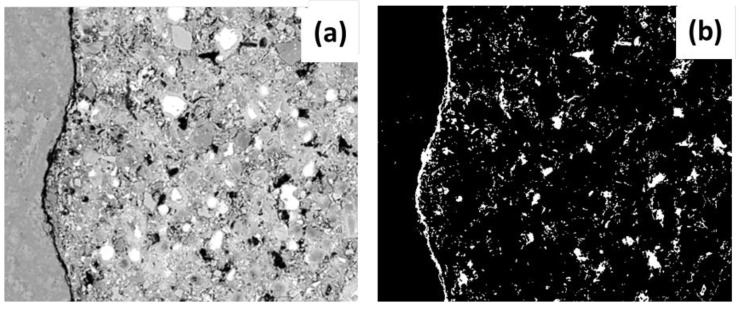
The total porosity of the image (**a**) initial image and (**b**) segmentation and binarization of image after application of threshold segregation.

The criterion of segregation or “threshold” was applied to the initial images of the study in order to know the values of porosity; this allowed sectioning of a specific range of tones in the gray scale—which can have values of between 0 and 255—while ensuring that this range of values was dependent on the materials. A histogram was used to determine the range of application (a pixel-frequency graph vs the value on the grayscale), encompassing the pixels included in the range of values from the initial (equal to zero) to the decrease in the last of the histogram frequency curves. The range of pixels from 0 to 75 (threshold) was chosen for the NCA samples; from 0 to 50 was chosen for the elements with RCA and SF. Therefore, all the image areas containing pixels within these ranges were considered pores or gaps. Figure 6 shows the final binarized image (segmentation of pixels in only two tones: black and white) of this process.

**Figure 7:**
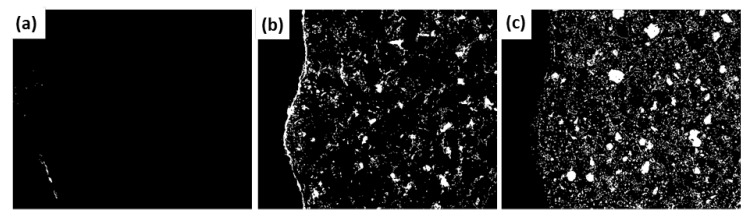
(**a**) Porosity of the aggregate, (**b**) porosity of the paste and (**c**) anhydrous material.

The multiplication operation with the coarse aggregate mask and the paste mask was applied to the binarized image, thereby obtaining the images that allowed the porosity identified in the aggregate (Figure 7a) and in the paste (Figure 7b) to be observed and quantified. Additionally, by applying a threshold (range 225–255) the elements that did not react chemically (anhydrous material) could be identified; in the (SEM-EDS) mapping of elements, they are shown as white pixels with a single basic chemical element (Figure 7c). Finally, once the porosity and the anhydrous material had been identified it was possible to obtain the percentage of hydration products; this is the result of removing the previous sum of porosity and anhydrous material from the original image.

**Figure 8:**
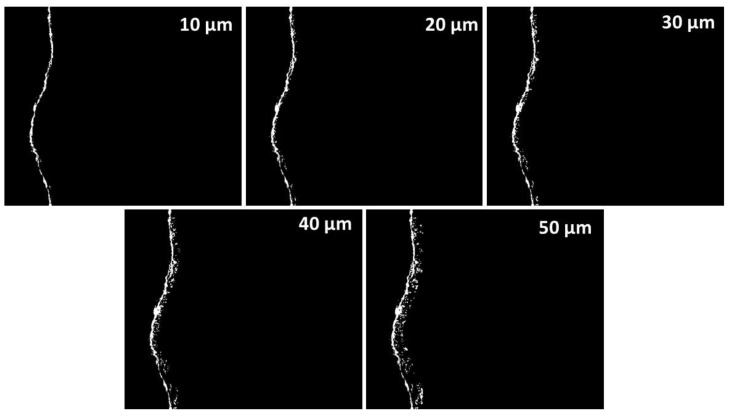
Images resulting from the sectors of the ITZ with the porosities determined at different theoretical thicknesses.

After these procedures and the image calibration, the sector in the scope of the ITZ was built (theoretical ITZ thickness located between the aggregate and paste throughout all areas). To do so, the sector previously established as the filter mask was used to establish the (theoretical) ITZ thicknesses of 10, 20, 30, 40 and 50 μm [14,27]. The image process used to achieve this was “dilation of objects”, applied to the original image of the aggregate mask and establishing the various thicknesses within the reach of the ITZ as “desired” (measured in pixels). If the pixel measurements were known it was possible to establish the thickness with the desired distance. Finally, this was overlaid onto the original binarized image. The results of the previous process that determined the sectors of ITZ can be seen in Figure 8. These were ultimately used as a mask to establish the percentage (histogram of the image) of porosity in the ITZ.

## 3. Results and Discussion

### 3.1. Mechanical and Physical Properties of the PC 

The results of the tests are shown in Table 5, where it can be seen that the reference concrete (NCA 100 C) had the best mechanical properties in terms of compression and flexure (21.9 and 4.9 MPa respectively). It was also observed that the inclusion of RCA in PC causes a reduction in its mechanical properties of 31% and 30% (compression and flexure respectively) with regard to NCA 100 C. Finally, it was established that the PC with SF did not generate increases in the mechanical properties. This may be because the SF does not react with the CH generated during the cement hydration, as due to the high porosity of this type of cement they were dissolved in the curing water [60].

The permeability coefficients established that there was a connection between the mechanical properties studied. This can be explained by the frequent association of permeability and porosity with the mechanical properties of concrete [61]. The PC (NCA 100 C) showed the lowest permeability coefficient in comparison with the other variables. This indicates that it has lower porosity and is, therefore, more capable of attaining better mechanical properties.

### 3.2. Mechanical and Physical Properties of the PC 

**Figure 9:**
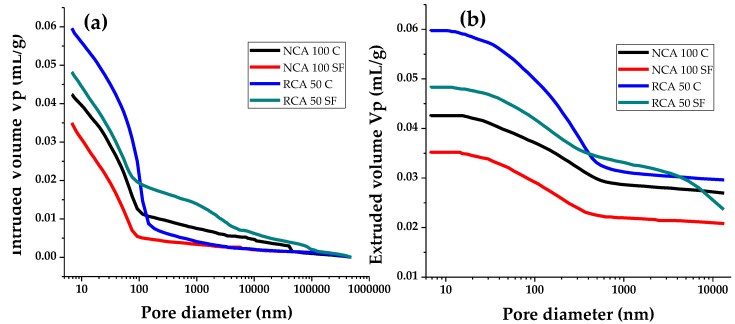
Mercury intrusion porosimetry, (**a**) intrusion and (**b**) extrusion.

Figure 9a shows the results obtained from the MIP tests in the enrichment phase (positive intrusion). The curves or isotherms of this graph show that their form is similar in all cases, their path being as follows: starting with slight increases from a common origin (the theoretical diameter of maximum pores φ_p max_ = 500,000 nm, macropores [62,63]), they continue to an abrupt inflexion point, located in the range of critical pore diameter 60 ≤ φ_pc_ ≤ 140 nm. Finally, they continue to an accelerated increase in the intrusion volume, which causes a steep rise until the points of maximum intrusion value are reached, minimum pore diameter φ_p min_ in the mesopore zone.

The PC sample—NCA 100 C—establishes the most representative pore samples as 7 ≤ φ_p_ ≤ 110 nm, with its inflexion point located in Vp = 0.01 mL/g. This means that a lower amount of Hg is needed to fill the large diameter (100 ≤ φ_p_ ≤ 400,000 nm) pores. In contrast, the NCA 100 SF sample shows fewer pores, which are significant in the size range of 6 ≤ φ_p_ ≤ 90 nm; the reason being the insertion of SF, as this is made up of small particles and may be used as a ‘filler’ for pores in the microstructure of concretes [64]. The RCA 50 C version establishes the highest number of pores of all the samples analyzed—set at 30 ≤ φ_p_ ≤ 200 nm—due to the inclusion of the RA; as this material is classified as low density and high porosity [1] the Hg can pass easily through its porous structure. Finally, it can be seen that adding SF to a sample of PC with RCA (RCA 50 SF) causes a reduction in the number of pores of 6 ≤ φ_p_ ≤ 80 nm; the cause is similar to that of the NCA 100 SF, although on this occasion with less importance (porous RCA).

To sum up, the order of the increase in porosity of PC with NCA was as follows: first NCA 100 SF and then NCA 100 C; regarding the PC with RA, first RCA 50 SF and finally RCA 50 C. The second group is affected by the RA with high original porosity. In both groups, the inclusion of SF (filling effect) is connected to the reduction of porosity.

The extrusion curves in Figure 9b help to interpret the morphology of the “trapped porosity”, identified when the intrusion–extrusion gaps were compared (a hysteresis phenomenon or the ability of the material to keep to the initial intrusion curve line as the Hg pressure returns to its initial state in the test). The difference between both curves established the quantity of Hg retained in the sample, a consequence of the number of bottleneck pores in the system [65,66]. The extrusion curves shown in the test samples have similar lines and the same relative positioning on the intrusion curves. Table 6 shows the values of trapped porosity (P_a_) obtained after determining the difference in volumes between both curves. It can be seen that the samples with RCA have twice as many bottleneck pores as the NCA samples. This is understandable if we consider that the RCA has open pores which are partially blocked by the new CP cement paste and that the use of C or SF is not significant in either sample group.

Table 6 and Table 7 show the values of the porous network indicators determined according to the previously established procedures [51,67,68]: maximum pore radius (r_max_), minimum radius (r_min_), critical radius (r_c_), average radius (r_m_), total pore area (A_tp_), intruded and extruded volume (V_i_ y V_e_) and P_a_. Vi = Volume intruded, Ve = Volume extruded. 

Regarding the r_max_ in the intrusion stage, the average radius established in the study samples (244,802 nm) highlights the ability of the PC to cause large pores—up to 40 times bigger than those noted in the usual recycled concretes [51,69]. However, in the extrusion stage, the average (6695 nm) is similar to the usual recycled concretes in their intrusion stage. In terms of the varieties studied, the order of radii variation between them is not considered important (less than 1% in both stages of the test). In both stages of the test, and all the varieties studied, r_min_ is considered a constant (equal to 3.4 nm). This raises two points for consideration: the MIP technique may be able to establish the network of pores in the micropore zone, and this minimum value probably has more in common with the limits or parameters of experimental techniques than with the varieties of this research.

The average values attained for the varieties studied in the case of r_c_ were 110.5 and 235.4 nm (intrusion and extrusion respectively). However, unlike the previous indicators (in the intrusion stage) the NCA 100 SF sample established an r_c_ some 50% less than that of the NCA 100 C, which shows that the use of SF is able to shift the r_c_ toward lower r_c_ values (closing the porous network). With regard to the RCA 50 C and RCA 50 SF versions (intrusion stage), the r_c_ variation between them is not important (a percentage of 50% SF is not important, the change of effect in r_c_ requires a high percentage of SF to be noticeable). Finally, the effect of the type of aggregate establishes that the use of RCA regarding NCA (intrusion stage) causes the former to establish a greater r_c_ than the latter (about 60% more), a result of the high porosity of the RCA itself. Concerning the extrusion stage, the previous trends have the same application, although the values established are greater than in the intrusion stage.

Regarding the indicators of r_m_, V_i,e_, and A_tp,_ the values shown in both stages of the study have, in percentage terms, similar interpretations to those established for r_c_; it can be seen that these are the most appropriate and sensitive for establishing the correlation between the study varieties and the porous network produced in those PCs.

### 3.3. Porosity, Anhydrous Material and Hydration Products in Cement Paste by mMeans of IA-SEM

**Figure 10:**
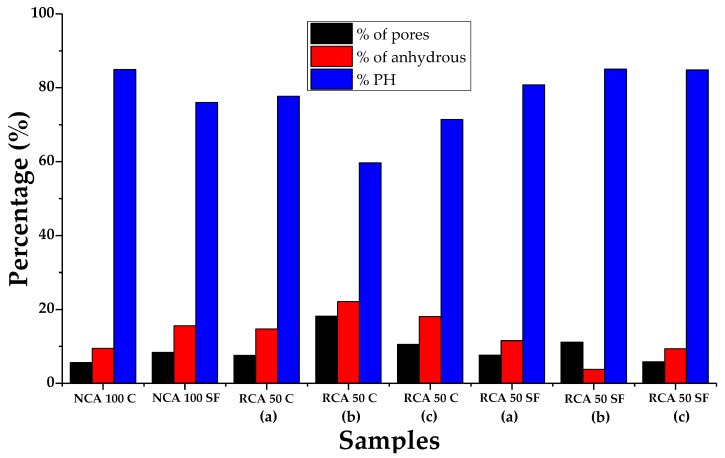
Porosity, anhydrous material and pH of the cement paste in the PC.

After performing an IA-SEM on the study samples, it was possible to obtain the percentages of porosity, anhydrous material and hydration products (PH) (see Figure 10). Three specifically different zones of interest were also evaluated for the RCA 50 C and RCA 50 SF samples: (a) ITZ between the new aggregate and the new paste, (b) ITZ between the old aggregate and the old paste and (c) ITZ between the old paste and the new paste.

As for porosity, and by comparing the samples that analyze the ITZ, it can be seen that this is higher in the samples containing 100% CP (possible paralysis of the hydration process); in the case of SF it is prolonged with age reducing the porosity, or is favored by the filling effect of pores which it causes. Among the zones of interest, b) (ITZ between old aggregate and old paste) establishes the greatest porosity (the variety linked to the less dense and more porous RCA concrete). If the use of RCA in the PC is eliminated (NCA 100 C and NCA 100 SF), it can be seen that there are minor variations of porosity between them, with the former being the best (in this case the filling effect of pores by SF is not favored, as the use of NCA does not include them). With regard to the anhydrous material and the PH, the use of SF establishes low percentages of anhydrous material and high ones of PH in the samples that contain RCA (the SF reacts with the CH present in the cement hydration to form more SCH). The sample RCA 50 SF is in the zone of interest b), the best of all (possibly due to the prolonged hydration over time of the old concrete from which the RCA originated). For the samples that use NCA, the use of C in the PC increases the formation of PH (the use of SF does not favor the creation of more PH, due to the possible effects of a blockage in hydration caused by a lack of pores or spaces wherein crystals may form).

### 3.4. Evaluation of Porosity at Different ITZ Thicknesses by Means of IA of SEM

**Figure 11:**
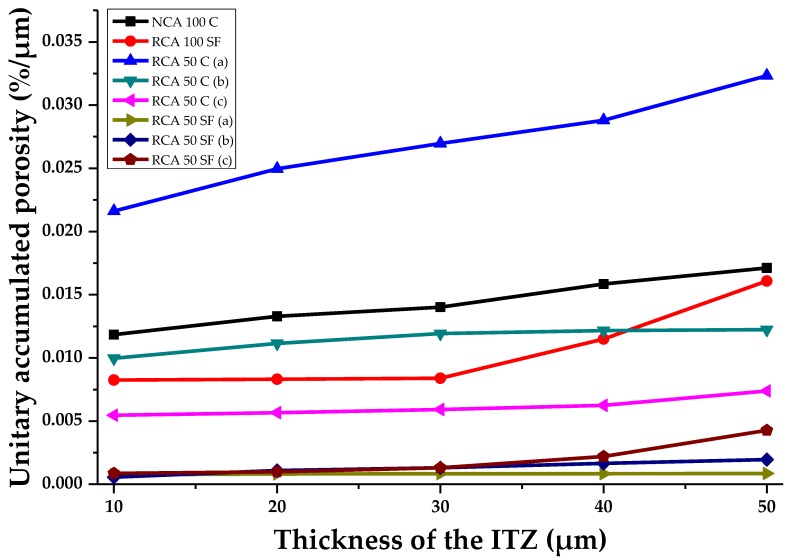
The porosity of the samples analyzed in the ITZ with several theoretical thicknesses.

Figure 11 shows the number of pores (unitary accumulated porosity, µm) determined in the study samples. It also considers the different theoretically possible thicknesses of the aggregate ITZ (ITZ of 10, 20, 30, 40 and 50 μm), which allows the transition from the porous network of the PC to be verified. It can be seen that there is a direct connection in most of the samples between the increase in the percentage of porosity and the increase in the theoretical ITZ thickness. The theoretical thickness is 10 μm, which establishes the best percentage of porosity compared with the other thicknesses evaluated; this confirms that the percentage of porosity of the ITZ decreases as its theoretical thickness increases. Another way to understand the curves is that the ITZ has finished when the curves of the samples tend toward a horizontal asymptote, with a constant—or non-increasing—percentage of porosity equal to that of the paste zone. Significantly, the three evaluated samples which had the lowest porosity in the ITZ are those with 50% of RCA and SF; this confirms that the use of SF leads to the filling effect in the ITZ porosity, making it denser and stronger. The RCA 50 C (a) sample shows the highest percentage of unitary porosity when the theoretical thickness is set at 10 μm (0.0216%/µm); at 50 μm reaches 0.032%/µm (the SEM image analyzed showed that this sample contained considerable porosity). This may have been due to the more porous nature of the RCA. Similarly, the effects of using SF were also observed in the NCA 100 SF, which had lower porosity than the NCA 100 C sample.

Therefore, it can be stated that the use of RCA in making a PC will cause increases in the ITZ porosity. However, the addition of SF may compensate for this, and achieve a denser cement matrix.

**Figure 12:**
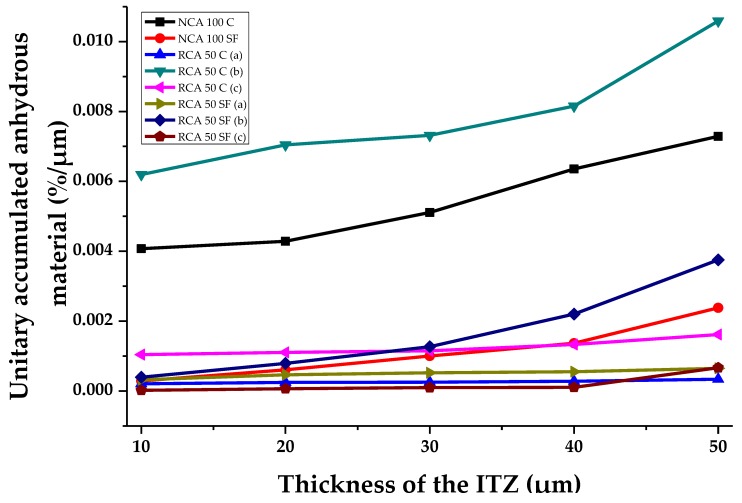
Anhydrous material of the samples analyzed in the IZT with various theoretical thicknesses.

Figure 12 shows the percentage of unitary accumulated anhydrous material at different theoretical ITZ thicknesses in the PC samples studied. The trend shows a direct, rising, connection between the increase of this parameter and the increase in the theoretical ITZ thickness. It should be noted that the samples with SF have lower percentages of anhydrous material. The SF can react with the cement hydration products and form new ones, which show low percentages of non-reacting material. The RCA 50 C sample (b), has the most anhydrous material: at 10 μm an important concentration of 0.0062%/µm can be seen, reaching 0.0106%/µm when the theoretical thickness is 50 μm. Additionally, comparing NCA 100 C and NCA 100 SF, the former is shown to contain a far greater percentage of anhydrous material at all theoretical ITZ thicknesses than the latter. Therefore, to sum up, it can be said that the use of SF contributes to the formation of more hydration products in the cement paste; this material can fill pores, thereby leading to lower percentages of anhydrous material.

**Figure 13:**
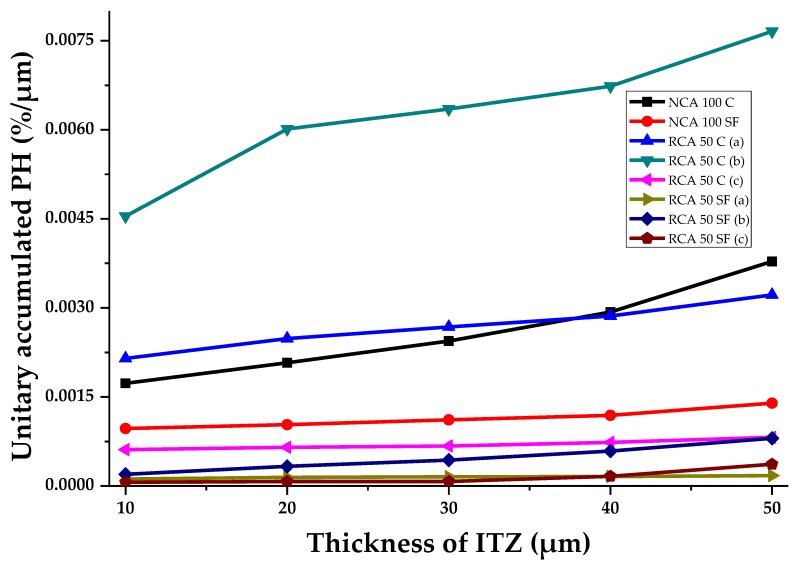
Hydration products of the samples analyzed in the IZT with various theoretical thicknesses.

The PH (the result of subtracting the porosity and the anhydrous material from the paste) that can be detected in the ITZ studied usually has a low value, as porosity dominates in an ITZ. Figure 13 shows the percentage of unitary pH at different theoretical ITZ thicknesses (%/µm). The RCA 50 C samples (a and b) have the highest percentage of PH, which corroborates previous paragraphs. This sample showed low percentages of porosity and anhydrous material. The NCA 100 C has a high amount of PH and shows a significant increase at theoretical ITZ thicknesses of between 40 and 50 μm.

The samples with SF (NCA 100 SF, RCA 50 SF (a and b)) have the lowest amounts of pH and correlate the results of the mechanical properties (they are the least resistant). Their mechanical properties were low compared with the samples made from Portland.

## 4. Conclusions

The physical properties evaluated show that using RA in the PC leads to a reduction in mechanical properties when compared to the reference PC (32%, 30% and 6% for compression, flexure and permeability, respectively), as these old aggregates are nearing the end of their life cycle and have less mechanical strength than natural aggregates. It is also shown that the use of SF in these concretes does not lead to an improvement in compressive strength, flexural strength or permeability, as they do not react with the CH crystals (which dissolve due to the high porosity of this concrete).

By means of image analysis with the NI Vision Assistant software, it was possible to prepare a script to make a mask of the aggregate and the cement paste. This was then used to analyze the percentage of porosity, the anhydrous material and the hydration products of the ITZ of several PC samples. This analysis confirmed that the PC with RA increased the porosity by 32% at 50 µm ITZ thickness between the new aggregate and the new paste. This is due to the RA being less dense, more porous and higher water-absorbent. It also shows that the use of SF caused an 89% decrease in porosity, mainly in the reference concrete (at 50 µm ITZ thickness in the new aggregate-new paste area), acting as a filling material for microporosity. The above information has shown that porosity increases when there is a thicker ITZ and that the most porous area of the PC is to be found between the new aggregate and the new paste. This method is an option for analyzing the microporosity of many materials. The evidence of the image analysis was corroborated by mercury intrusion porosimetry. The use of RCA in making permeable concrete leads to higher porosity in the cement paste and the ITZ; but the use of SF in the concrete also helps reduce the porosity, as it fills the gaps in the microstructure.

## 5. Future Research 

To find a correlation of XRD with SEM to locate specific compounds and their relationship with mechanical behavior.

To obtain a numerical quantification of the amount of old mortar adhered to the recycled aggregates and its connection with other mechanical and durability properties.

Regarding the technique of image analysis, there are parameters that will be necessary to calibrate in the definition of these masks; among the most complex and therefore the most demanding future study due to its great diversity of application alternatives are morphological filtering and noise filtering.

The use of scripts for image analysis can be used to measure porosities and failure sites (cracking) for a wide range of materials (ceramic, metallic and polymeric).

Images can be obtained in different layers and at different depths of the sample and develop scripts so that the material can be considered statistically homogeneous (the results are representative of the sample volume).

## Figures and Tables

**Table 1:** Properties of the aggregates.

Properties	NCA	RCA
Aggregate size (mm)	12.70	12.70
Concrete strength of origin (MPa)	-	23.00
Absorption (%)	1.67	6.28
Humidity (%)	1.67	1.80
Density (kg/cm^3^)	2.65	2.35
Porosity (%)	11.00	16.31
Rebound index (MPa)	33.20	29.90

**Table 2:** Chemical composition of Portland cement and silica fume.

Oxide (%)	SiO_2_	Al_2_O_3_	Fe_2_O_3_	CaO	SO_3_	K_2_O	Na_2_O	MgO
Cement	19.94	4.40	2.97	63.5	3.08	0.42	0.12	-
Silica Fume	95.22	0.08	2.37	0.26	0.11	0.56	0.30	0.24

**Table 3:** Properties of Portland cement and silica fume.

Material	Density (kg/m^3^)	Specific Surface (m^2^/kg)	Average Size (µm)
Cement	3150	1400	15–25
Silica Fume	2270	19,600	0.1–0.2

**Table 4:** Previous concrete mixtures for a specimen of 15 × 15 × 50 cm (0.0113 m^3^).

Materials	NCA 100 C	NCA 100 SF	RCA 50 C	RCA 50 SF
Water (L)	1.9781	1.9781	1.9467	1.9467
NCA (kg)	17.5568	17.5571	8.7785	8.7785
RCA (kg)	-	-	8.7785	8.7785
Cement (kg)	5.5621	5.0059	5.5621	5.0059
Silica fume (kg)	-	0.5562	-	0.5562

**Table 5:** Properties of PC.

Study Variables	Compression Strength (MPa)	Flexural Strength (MPa)	Water Permeability Coefficient (m/s)
NCA 100 C	21.9	4.9	8.9 × 10^−03^
NCA 100 SF	20.8	4.4	1.1 × 10^−02^
RCA 50 C	15.5	3.6	1.0 × 10^−02^
RCA 50 SF	15.1	3.4	1.1 × 10^−02^

**Table 6:** Porous network indicators in Hg extruded stage.

Sample	r_max_ (nm)	r_min_ (nm)	r_c_ (nm)	r_m_ (nm)	V_e_ (mL/g)	P_a_ (mL/g)
NCA 100 C	6708.05	3.4	273.50	90.918	0.0269	0.0154
NCA 100 SF	6730.10	3.4	121.25	64.522	0.0208	0.0144
RCA 50 C	6702.55	3.4	273.50	90.650	0.0296	0.0299
RCA 50 SF	6637.60	3.4	273.50	150.591	0.0236	0.0246

**Table 7:** Porous network indicators in Hg intruded stage.

Sample	r_max_ (nm)	r_min_ (nm)	r_c_ (nm)	r_m_ (nm)	V_i_ (mL/g)	A_tp_ (mL/g)
NCA 100 C	244,514.75	3.400	110.700	28.333	0.0424	4.15 × 10^02^
NCA 100 SF	246,402.35	3.400	57.600	18.522	0.0351	4.26 × 10^02^
RCA 50 C	242,670.09	3.378	136.691	39.307	0.0595	3.77 × 10^02^
RCA 50 SF	245,620.64	3.400	137.000	29.588	0.0482	7.41 × 10^02^
